# Exploring the effects of naringin on oxidative stress-impaired osteogenic differentiation via the Wnt/β-catenin and PI3K/Akt pathways

**DOI:** 10.1038/s41598-024-64952-2

**Published:** 2024-06-18

**Authors:** Hui Wang, Jun Liang, Yiran Wang, Junyuan Zheng, Ying Liu, Yiyang Zhao, Yixuan Ma, Pei Chen, Xufang Yang

**Affiliations:** 1 Department of Pathophysiology, Mudanjiang Medical University, No. 3 Tongxiang Road, Mudanjiang , 157011 Heilongjiang China; 2 Department of Morphology Laboratory, Mudanjiang Medical University, Mudanjiang, 157011 Heilongjiang China; 3 Department of Foreign Languages, Mudanjiang Medical University, Mudanjiang, 157011 Heilongjiang China; 4 The First Clinical Medicine College, Southern Medical University, Guangzhou, 510000 Guangdong China; 5 The First Clinical Medicine College, Harbin Medical University, Harbin, 150000 Heilongjiang China; 6 The first Clinical Medicine College, Mudanjiang Medical University, NO. 3 Tongxiang Road, Mudanjiang, 157011 Heilongjiang China

**Keywords:** Osteoporosis, Naringin, Oxidative stress, Wnt/β-catenin signaling pathway, PI3K/Akt signaling pathway, Stem-cell differentiation, Cell signalling

## Abstract

This study aimed to explore naringin’s potential to promote the osteogenic differentiation of MC3T3-E1 under oxidative stress. It delved into Nar’s connection with the Wnt/β-catenin and PI3K/Akt signaling pathways. Initially, 2911 OP-related genes were analyzed, revealing close ties with the PI3K/Akt and Wnt pathways alongside oxidative stress. Nar’s potential targets—ESR1, HSP90AA1, and ESR2—were identified through various databases and molecular docking studies confirmed Nar’s affinity with ESR1 and HSP90AA1. Experiments established optimal concentrations for Nar and H_2_O_2_. H_2_O_2_ at 0.3 mmol/L damaged MC3T3-E1 cells, alleviated by 0.1 µmol/L Nar. Successful establishment of oxidative stress models was confirmed by DCFH-DA probe and NO detection. Nar exhibited the ability to enhance osteogenic differentiation, counteracting oxidative damage. It notably increased osteoblast-related protein expression in MC3T3-E1 cells under oxidative stress. The study found Nar’s positive influence on GSK-3β phosphorylation, β-catenin accumulation, and pathway-related protein expression, all critical in promoting osteogenic differentiation. The research concluded that Nar effectively promotes osteogenic differentiation in MC3T3-E1 cells under oxidative stress. It achieved this by activating the Wnt/β-catenin and PI3K/Akt pathways, facilitating GSK-3β phosphorylation, and enhancing β-catenin accumulation, pivotal in osteogenesis.

## Introduction

The skeletal system plays a crucial role through structural support, organ protection, and facilitating movement in the body. Osteoblast-mediated bone formation and osteoclast-mediated bone resorption are key homeostatic processes that occur in adult bones to replace aging tissues and repair damage^1^. Owing to the rapid aging of populations worldwide, the prevalence of OP continues to rise. Therefore, the prevention and treatment of OP have become essential issues requiring urgent attention in modern medicine.

OP is mainly caused by abnormal bone resorption rather than formation^2^, whereby increases in bone resorption lead to the acceleration of bone loss. Therefore, inhibiting bone resorption or activating bone formation is an effective way to treat osteoporosis. In recent years, traditional Chinese medicines' prophylactic and therapeutic effects on OP have attracted widespread attention from researchers, especially since the active ingredients typically have few side effects and high cost and time efficiency^3^.

The accumulation of free radicals, such as or reactive oxygen species (ROS), is a major cause of OP-related morbidity^4,5^. ROS, as an important intermediate product of oxidative stress, are related to osteogenic differentiation and bone regeneration^6^. Low ROS levels play a role in regulating signaling mediators conducive for bone regeneration. In contrast, excess ROS production often suppresses Wnt/β-catenin signaling and activates FoxO signaling, resulting in impaired bone regeneration^7^.

In 2015, Nar was found to protect human adipose stem cells from osteogenic differentiation after hydrogen peroxide (H_2_O_2_)-induced injury^8^. Nar is a dihydroflavonoid widely found in *Urticaceae* plants and one of the most representative medicinal active substances derived from Rhizoma drynariae^9^. Various studies have elucidated the pharmacological properties of Nar, including anti-allergic^10^, anti-atherosclerotic^11^, antibacterial and anti-inflammatory^12^, anti-injury^13^, anti-arrhythmia, anti-tussive, anti-diabetic^14^, anti-OP^15^, anti-oxidant^16^, anti-arthritic^17^, and anti-osteonecrotic. A preclinical rat toxicology study evaluated the tolerability of Nar and demonstrated low toxicity and high safety^18^.

Nar-mediated promotion of osteogenic differentiation has been previously reported^19^, and Wnt/β-catenin and PI3K/Akt signaling pathways are known to be implicated in this process^20^. As both pathways can induce glycogen synthase kinase-3 β (GSK-3β) activation, an interaction between the two pathways is believed to exist. However, whether Nar can reverse the inhibitory effect of oxidative stress on osteogenic differentiation through cross-talk of the Wnt/β-catenin and PI3K/Akt signaling pathways has not been reported.

This study explored the molecular mechanism by which Nar promotes osteogenic differentiation by analyzing the expression of proteins related to the above two pathways and those involved in promoting osteogenic differentiation under oxidative stress to provide new targets for OP treatment.

## Results

### Screening OP-related DEGs and enrichment analysis

**Figure 1:**
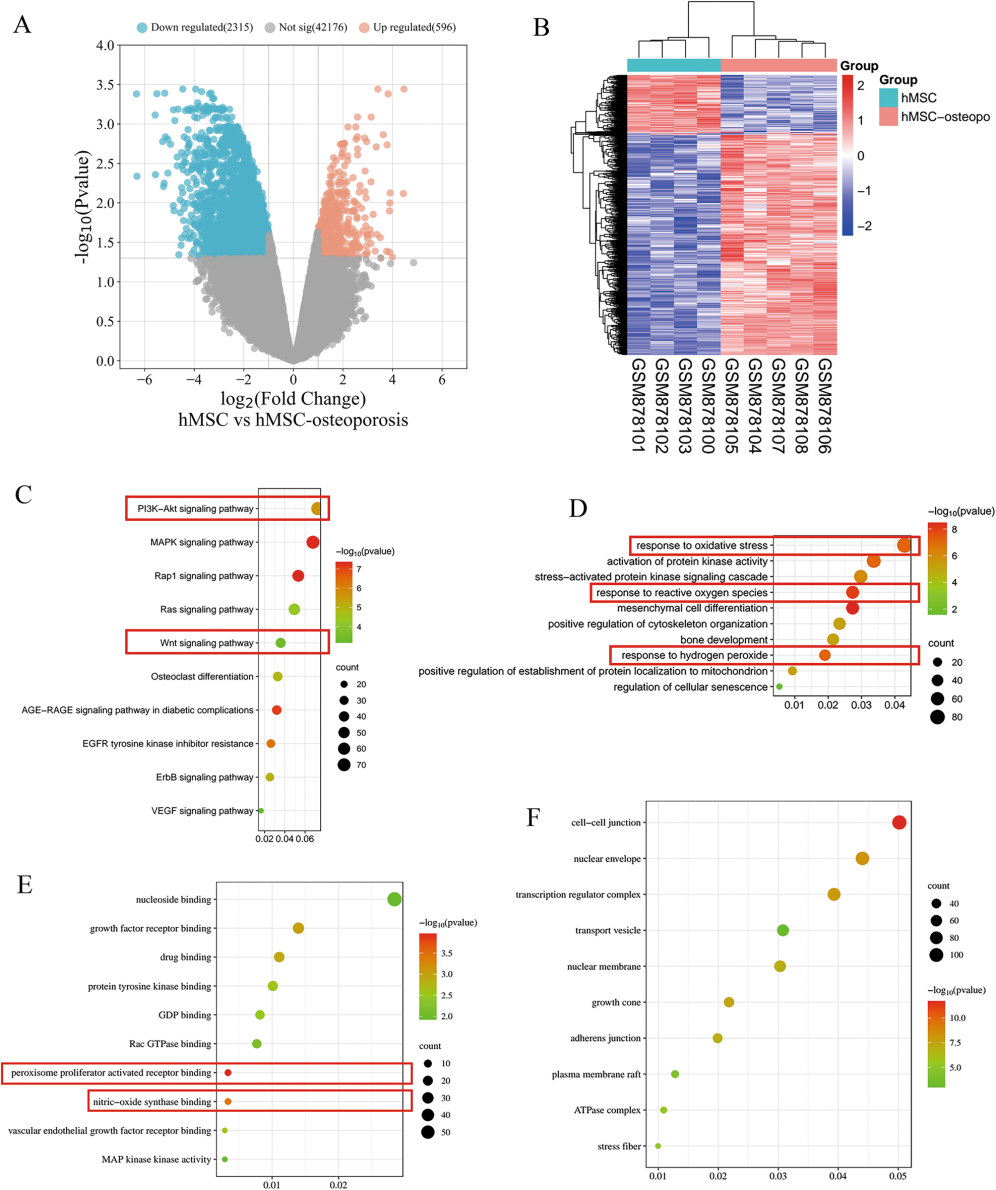
Screening and enrichment analysis of OP-associated DEGs. (**A**) A volcano map of OP-related DEGs (red dots represent high-expression DEGs, blue dots represent low-expression DEGs); (**B**) A OP-related DEG expression calorigram (x-axis represents sample name, y-axis represents gene name, red colour represents high differential gene expression, blue colour represents low differential gene expression).(**C**) KEGG enrichment analysis; (**D**–**F**) GO enrichment analysis with biological processes (**D**), molecular function (**E**), and cell component (**F**). The dot size represents the number of genes enriched, with a larger dot indicating higher enrichment, and the colour depth represents the *P* value, with a darker colour indicating a smaller *P* value.

The DEGs were screened using GEO2R standardized gene microarray data from the GEO database. Volcanic and heat map analyses revealed 2911 OP-related DEGs in the GSE35958 dataset, consisting of 596 upregulated and 2315 downregulated genes (Fig. 1A–B).

**Figure 2:**
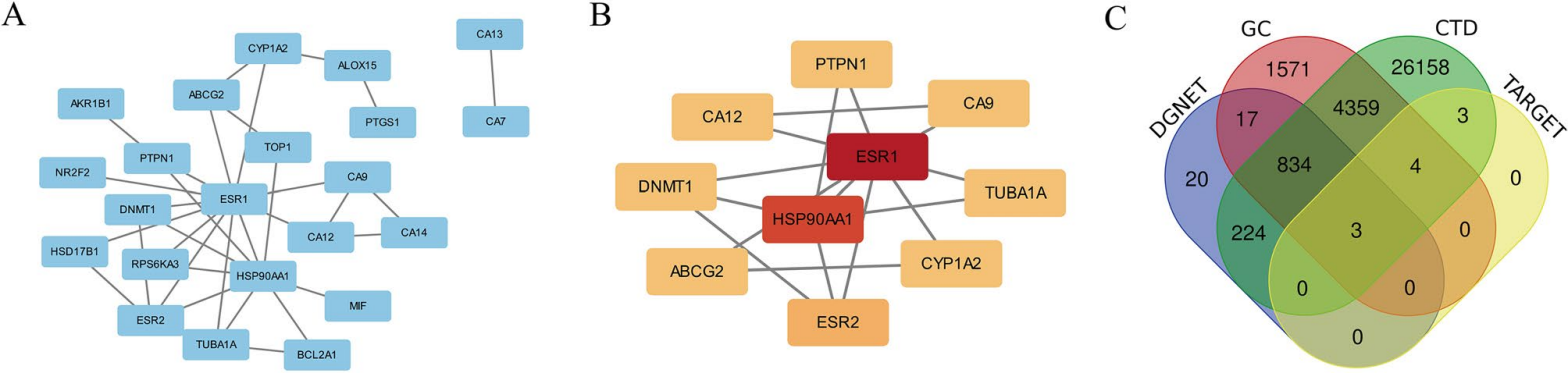
Potential Nar action targets associated with OP. (**A**) A PPI network diagram of Nar potential targets; (**B**) The top 10 core genes according to node degree values of the potential NAR targets (colour depth indicates the degree value, where the darker the colour, the greater the degree value); (**C**) Venn diagram of potential targets of Nar for OP treatment (*DGNET* DisGeNET, *GC* GeneCards, *CTD* comparative toxicogenomics database, *TARGET* Nar core target).

The top 10 results of the KEGG and GO enrichment analyses were plotted as bubble maps to understand further the enrichment pathways and functions of the OP-related DEGs. The KEGG results showed that the DEGs were mainly enriched in the PI3K/Akt, MAPK, and Wnt signaling pathways (Fig. 1C). The GO analysis results showed that (1) BP mostly included reactions to oxidative stress, ROS, and H_2_O_2_ (Fig. 1D); (2) MF mostly included binding with growth factor receptors, peroxisome proliferator-activated receptors, and NO synthase (Fig. 1E); and (3) CC mostly included intercellular junctions, nuclear envelope, and nuclear membranes (Fig. 1F).

### Screening of OP-related potential Nar action targets

To explore the potential targets of Nar associated with OP, a PPI network with 29 potential targets was established using the SMILES chemical formula from the PubChem website as a keyword in TargetNet, inputting them into the STRING database, and delimiting the species as Homo sapiens. Further visualization was performed using Cytoscape software (Fig. 2A). Ten core targets (ER1, HSP90AA1, ESR2, ABCG2, DNMT1, CA12, PTPN1, CA9, TUBA1A, and CYP1A2) were obtained and ranked from largest to smallest degree value (Fig. 2B). Using the keyword “osteoporosis”, 31,585 OP-related proteins were obtained from the CTD database, 1098 OP-related proteins were obtained from DisGeNET, and 6788 OP-related proteins were obtained from GeneCards. A Venn diagram was constructed using an online tool, and the intersection sets between the OP-related proteins and Nar core targets were obtained from the three databases above. In total, 3 potential targets for OP-related drugs (ESR1, HSP90AA1 and ESR2) were obtained (Fig. 2C).

### Molecular docking and interaction analysis of Nar with ESR1 and HSP90AA1

**Figure 3:**
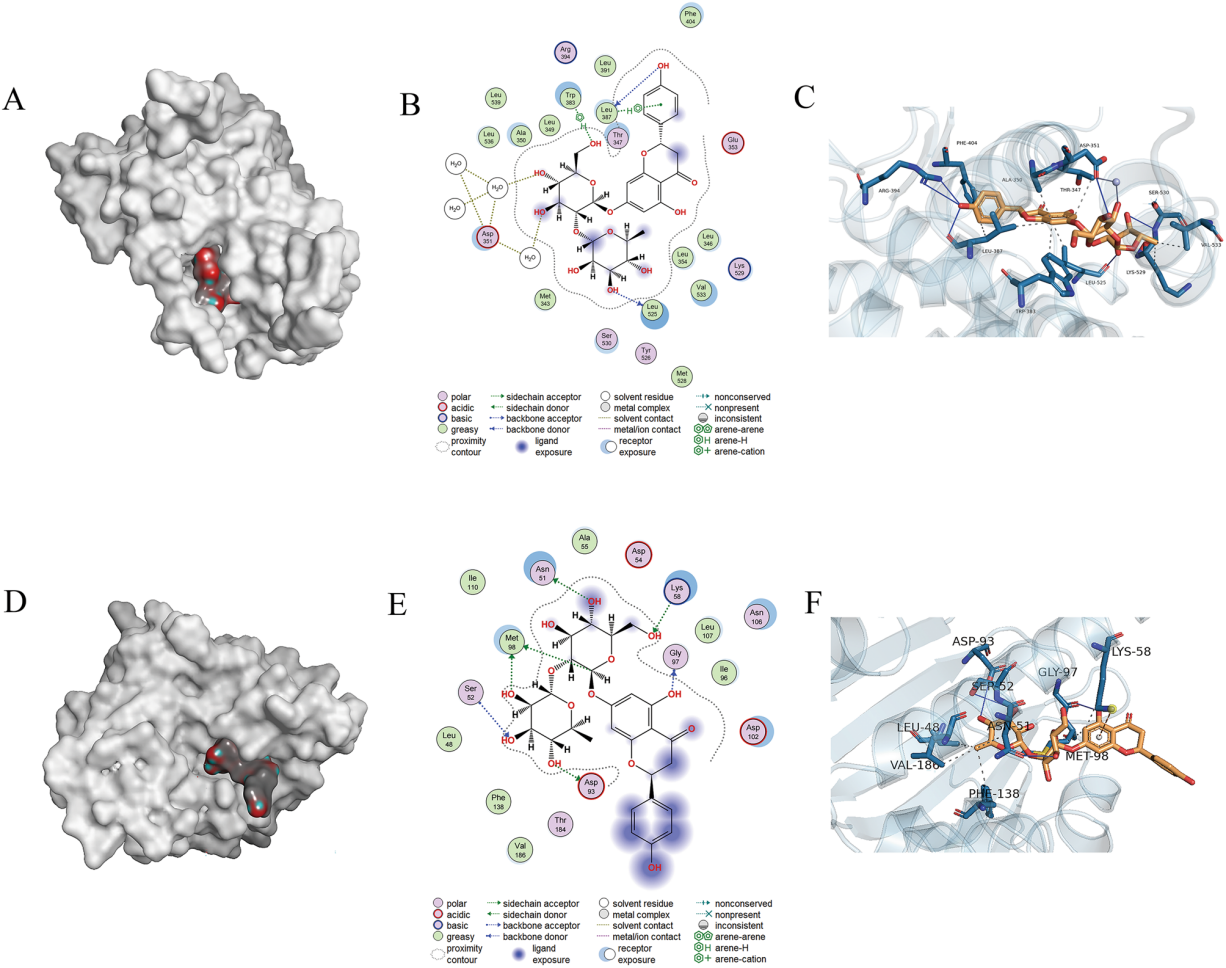
Molecular docking and interaction of Nar with ESR1 and HSP90AA1. (**A**) A 3D view of the protein–ligand complex of ESR1 and Nar; (**B**) A 2D view of the interaction between ESR1 and Nar; (**C**) A 3D view of ESR1 and Nar residue interactions; (**D**) A 3D view of the protein–ligand complex of HSP90AA1 and Nar; (**E**) A 2D view of the interaction between HSP90AA1 and Nar; and (**F**) A 3D view of HSP90AA1 and Nar residue interactions.

To further clarify the interaction between Nar and its potential disease-related targets, molecular pairing results revealed that Nar was tightly bound to ESR1 and HSP90AA1, and that the protein–ligand complex was relatively stable (Fig. 3A,D). The two-dimensional analysis showed hydrogen bonds with water bridges between Nar and ESR1 and HSP90AA1 (Fig. 3B,E). Interaction analysis between Nar and ESR1 showed that Nar formed hydrophobic interactions with Thr347, Ala350, Trp383, Leu387, Phe404, Leu525, Lys529, and Val533. In addition, Nar interacted with Asp351, Leu387, Arg394, Leu525, and Ser530 via hydrogen bonding, whereas Nar interacted with Asp351 via a water bridge (Fig. 3C). Interaction analysis between Nar and HSP90AA1 revealed that Nar formed hydrophobic interactions with Leu48, Asn51, Lys58, Met98, Phe138, and Val186. In addition, Nar interacted with Asn51, Ser52, Asp93, and Gly97 via hydrogen bonding, and Lys58 via cation interaction (Fig. 3F). These results showed that Nar had a high affinity for ESR1 and HSP90AA1, confirming their usefulness as potential targets for Nar.

### Nar alleviates the effect of H_2_O_2_ on MC3T3-E1 cell viability

**Figure 4:**
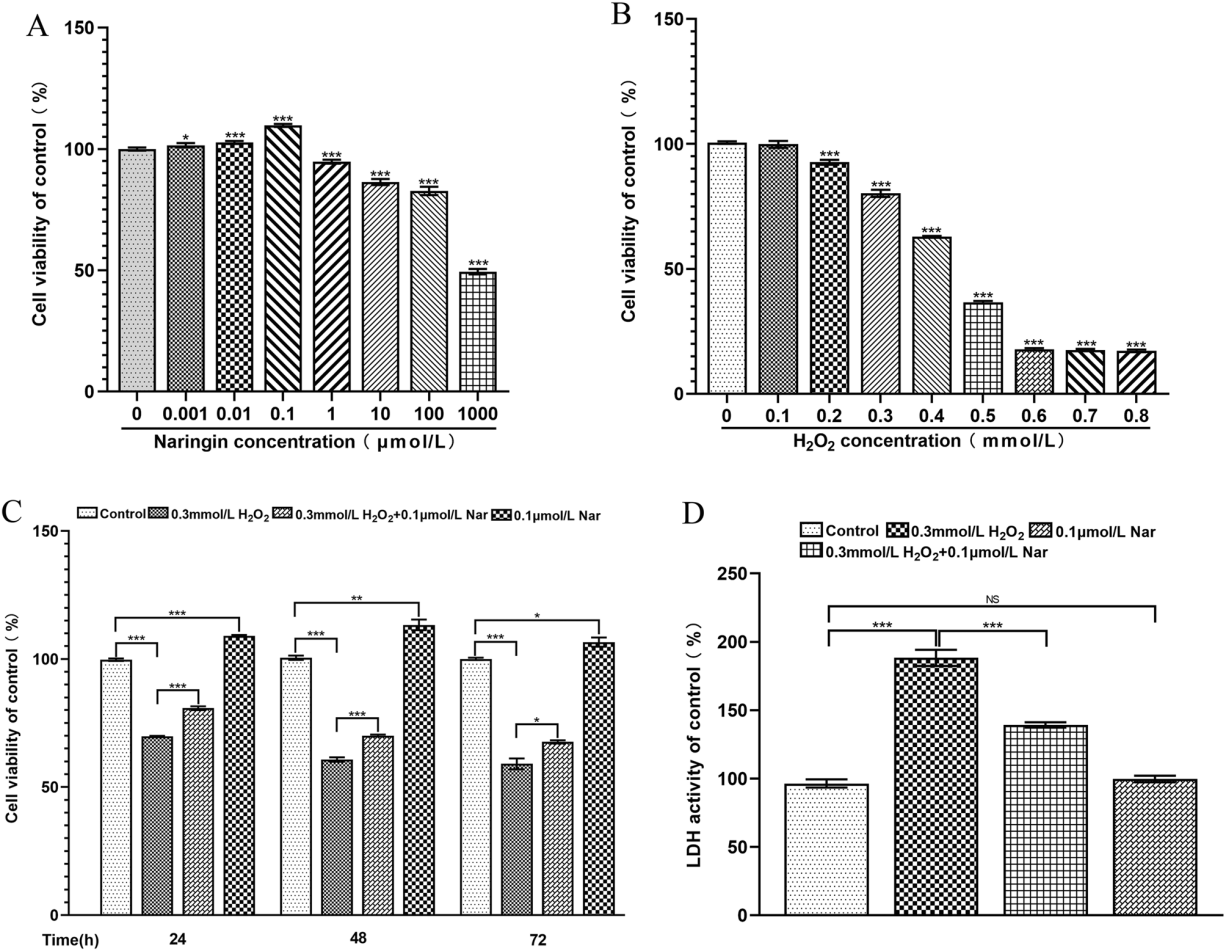
Effect of Nar and H_2_O_2_ on MC3T3-E1 cell viability. (**A**) The effect of different concentrations of Nar on MC3T3-E1 cell viability; (**B**) The effect of different concentrations of H_2_O_2_ on MC3T3-E1 cell viability; (**C**) The effects of 0.3 mM H_2_O_2_ and 0.1 µmol/L Nar co-treatment on the viability of MC3T3-E1 cells; (**D**) The effect of 0.3 mM H_2_O_2_ and 0.1 µM Nar on the release of LDH by MC3T3-E1 cells. *P < 0.05, **P < 0.01, ***P < 0.001 (n = 4).

To further determine the interaction between NAR and the potential disease-related targets, ESR1 and HSP90AA1, the CCK-8 method was used to detect different concentrations of Nar (0.001 μM, 0.01 μM, 0.1 μM, 1 μM, 10 μM, 100 μM, and 1000 mM) and H_2_O_2_ (0.1 mM, 0.2 mM, 0.3 mM, 0.4 mM, 0.5 mM, 0.6 mM, 0.7 mM, and 0.8 mM) on the MC3T3-E1 cells. The results showed that the activity of MC3T3-E1 significantly improved following administration of 0.1 μM Nar (Fig. 4A), but decreased significantly after 24 h treatment with 0.3 mM H_2_O_2_, with an overall survival rate of MC3T3-E1 cells of 75% (Fig. 4B). Thus, 0.1 µM Nar and 0.3 mM H_2_O_2_ were optimal concentrations (*P* < 0.05). Following treatment with 0.3 mM H_2_O_2_ for 24, 48, and 72 h, cell viability of MC3T3-E1 cells was significantly restored after the addition of 0.1 μM Nar (Fig. 4C). Similar results were obtained by detecting LDH release after 24 h of the combined incubation (Fig. 4D), suggesting that Nar could reduce the impact of H_2_O_2_ on MC3T3-E1 cell viability. The differences between all groups were statistically significant (*P* < 0.05).

### Nar reduced H_2_O_2_-induced oxidative stress in MC3T3-E1 cells

**Figure 5:**
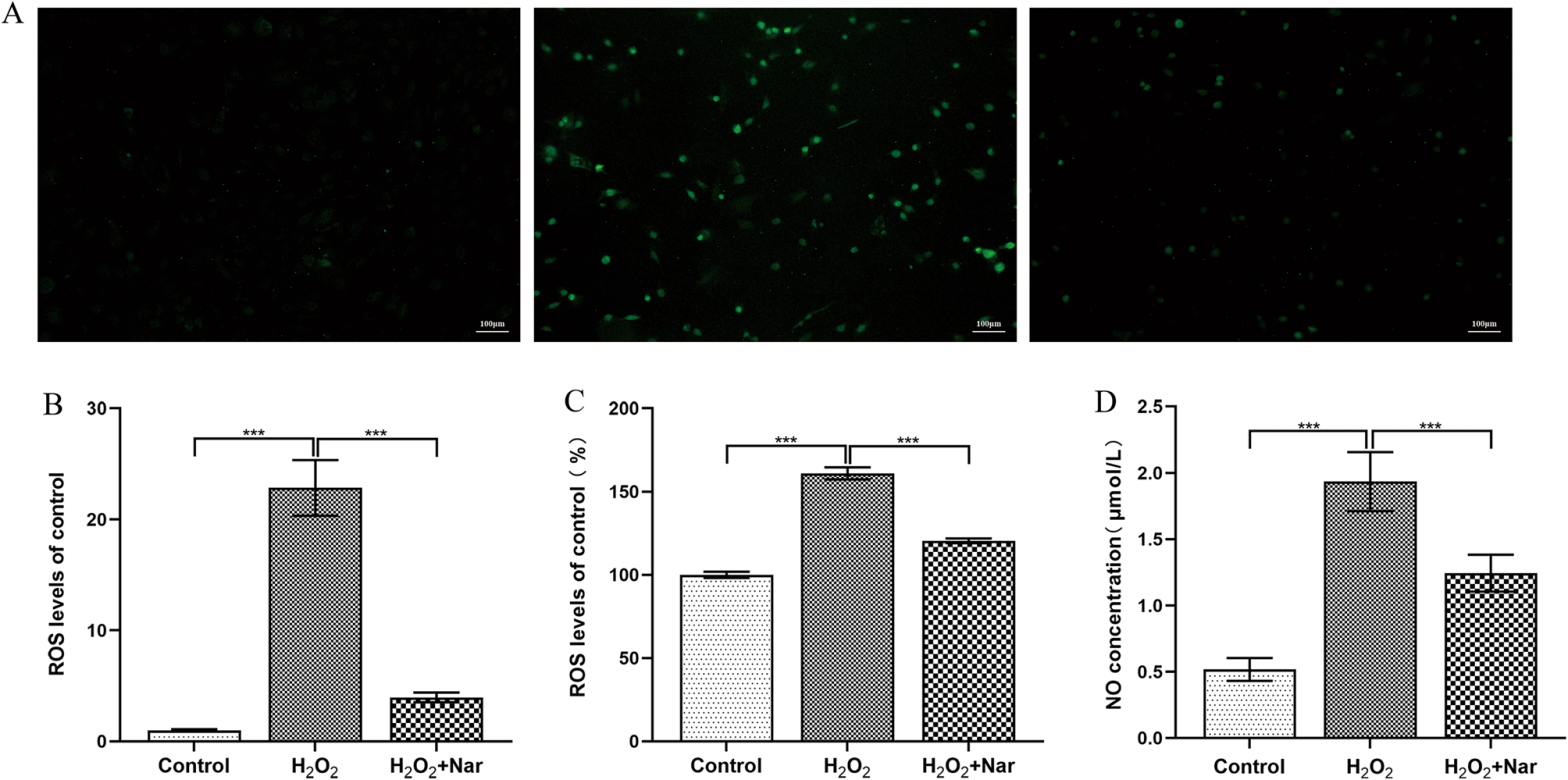
Effect of Nar on H_2_O_2_-induced oxidative stress in MC3T3-E1 cells. (**A**) ROS fluorescence intensity changes in each group (100 ×); (**B**) ROS fluorescence intensity changes in each group; (**C**) ROS absorbance changes in each group; (**D**) Changes in NO production in each group. ***P < 0.001 (n = 4).

To explore the effect of Nar on the oxidative stress in MC3T3-E1 cells induced by H_2_O_2_, a DCFH-DA fluorescent probe was used to detect intracellular ROS levels. Fluorescence microscopy revealed that MC3T3-E1 cells treated with H_2_O_2_ had the highest fluorescence intensity, which decreased after Nar treatment (Fig. 5A,B), indicating that Nar could reduce H_2_O_2_-induced oxidative stress in MC3T3-E1 cells. Similar results were obtained in assays evaluating intracellular ROS and NO production using an enzyme labeling instrument (Fig. 5C,D). The differences between all groups were statistically significant (*P* < 0.001).

### Effect of Nar on osteogenic differentiation of MC3T3-E1 cells following H_2_O_2_ injury

**Figure 6:**
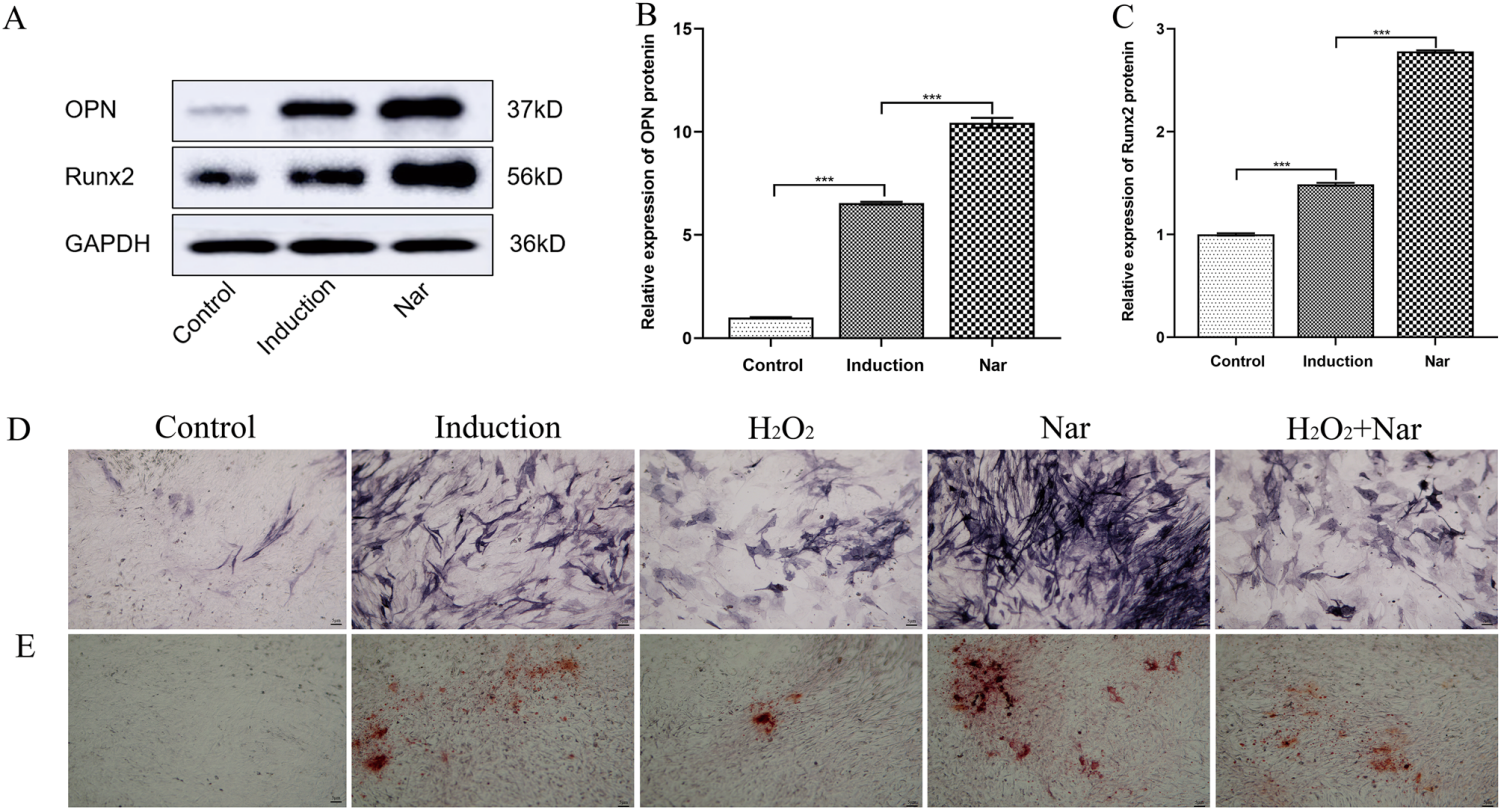
Effect of Nar on osteogenic differentiation of MC3T3-E1 cells following H_2_O_2_ injury. (**A**) Protein expression in each group; (**B**) Relative expression of OPN; (**C**) Relative expression of Runx2; (**D**) ALP staining 2 weeks after osteogenic differentiation (100 ×); (**E**) Alizarin Red S staining after 4 weeks of osteogenic differentiation (100×). ***P < 0.001.

To determine the effect of Nar on the osteogenic differentiation of MC3T3-E1 cells, the expression of OPN and Runx2 was detected 14 days after osteogenic induction. Protein expression increased more than that of the control, indicating successful induction. After administration of Nar, the expression of OPN and Runx2 significantly increased compared to that in the induction group, indicating that Nar can promote the osteogenic differentiation of MC3T3-E1 cells (Fig. 6A–C).

To further determine the effect of Nar, ALP, and Alizarin Red S staining were performed 2 and 4 weeks after osteogenic induction, respectively. Compared with the control group, ALP staining in the induction group was purple and positive. In contrast, the H_2_O_2_ group was lighter than the induction group, and the Nar group was strongly positive, indicating that Nar ameliorated oxidative inhibition after oxidative damage to some extent (Fig. 6D). Alizarin Red S staining showed that calcium salt deposition in the outer interstitial cells in each group was consistent with the results of ALP staining (Fig. 6E), indicating that Nar promoted bone formation in MC3T3-E1 cells under oxidative stress. The differences between all groups were statistically significant (*P* < 0.001).

### Nar promotes osteogenic differentiation of MC3T3-E1 cells via the Wnt/β-catenin signalling pathway under oxidative stress

**Figure 7:**
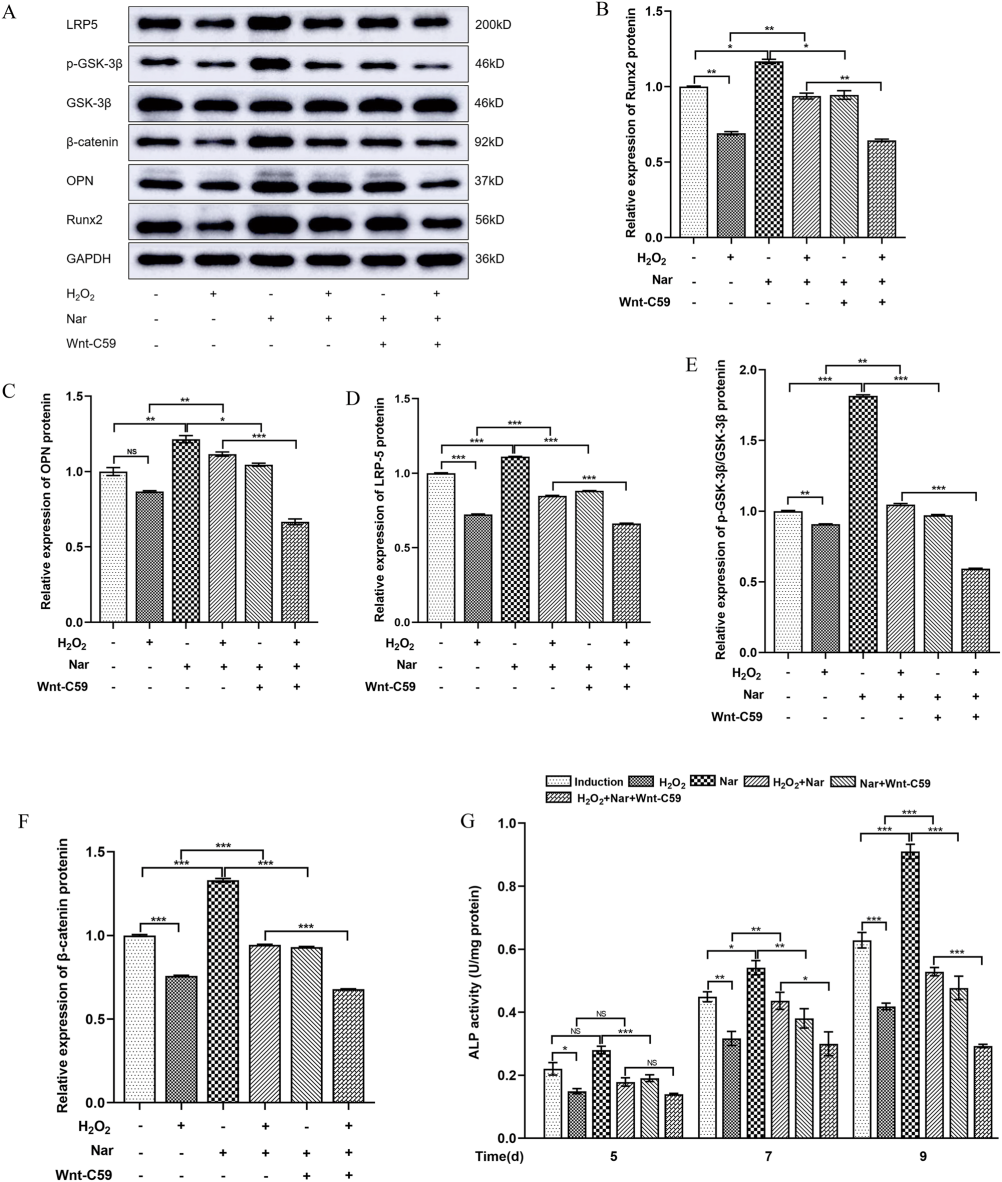
Relationship between Nar-mediated Wnt/β-catenin signalling pathway and osteogenic differentiation of MC3T3-E1 cells under oxidative stress. (**A**) Protein expression in each group; (**B**–**F**) Relative expression of Runx2 (**B**), OPN (**C**), LRP-5 (**D**), p-GSK-3β/GSK-3β (**E**), and β-catenin (**F**). (**G**) ALP activity in each group. *P < 0.05, **P < 0.01, ***P < 0.001 (n = 4).

To investigate the relationship between the Nar-mediated Wnt/β-catenin signalling pathway and osteogenic differentiation of MC3T3-E1 cells under oxidative stress, changes in pathway-related and osteogenic proteins in each group were detected. Compared to the induction group, both H_2_O_2_ group protein expression levels decreased, whereas these increased in the Nar group. This suggests that H_2_O_2_ inhibited and Nar promoted osteogenic differentiation through the Wnt/β-catenin signaling pathway (Fig. 7). Compared to the H_2_O_2_ group, the expression of pathway-related proteins in the H_2_O_2_ + Nar group also increased. Moreover, after administration of Wnt-C59, a Wnt/β-catenin signaling pathway inhibitor, the expression of pathway-related proteins and osteogenic effect decreased in the Nar group, compared to controls. These results suggested that Nar could promote osteogenic differentiation of MC3T3-E1 cells through Wnt/β-catenin signaling pathway under oxidative stress (Fig. 7). Intracellular ALP activity was measured at 5, 7, and 9 days after osteogenic induction and the results were consistent with the protein expression levels (Fig. 7G). The differences between all groups were statistically significant (*P* < 0.05).

### Nar promotes osteogenic differentiation of MC3T3-E1 cells via the PI3K/Akt signalling pathway under oxidative stress

**Figure 8:**
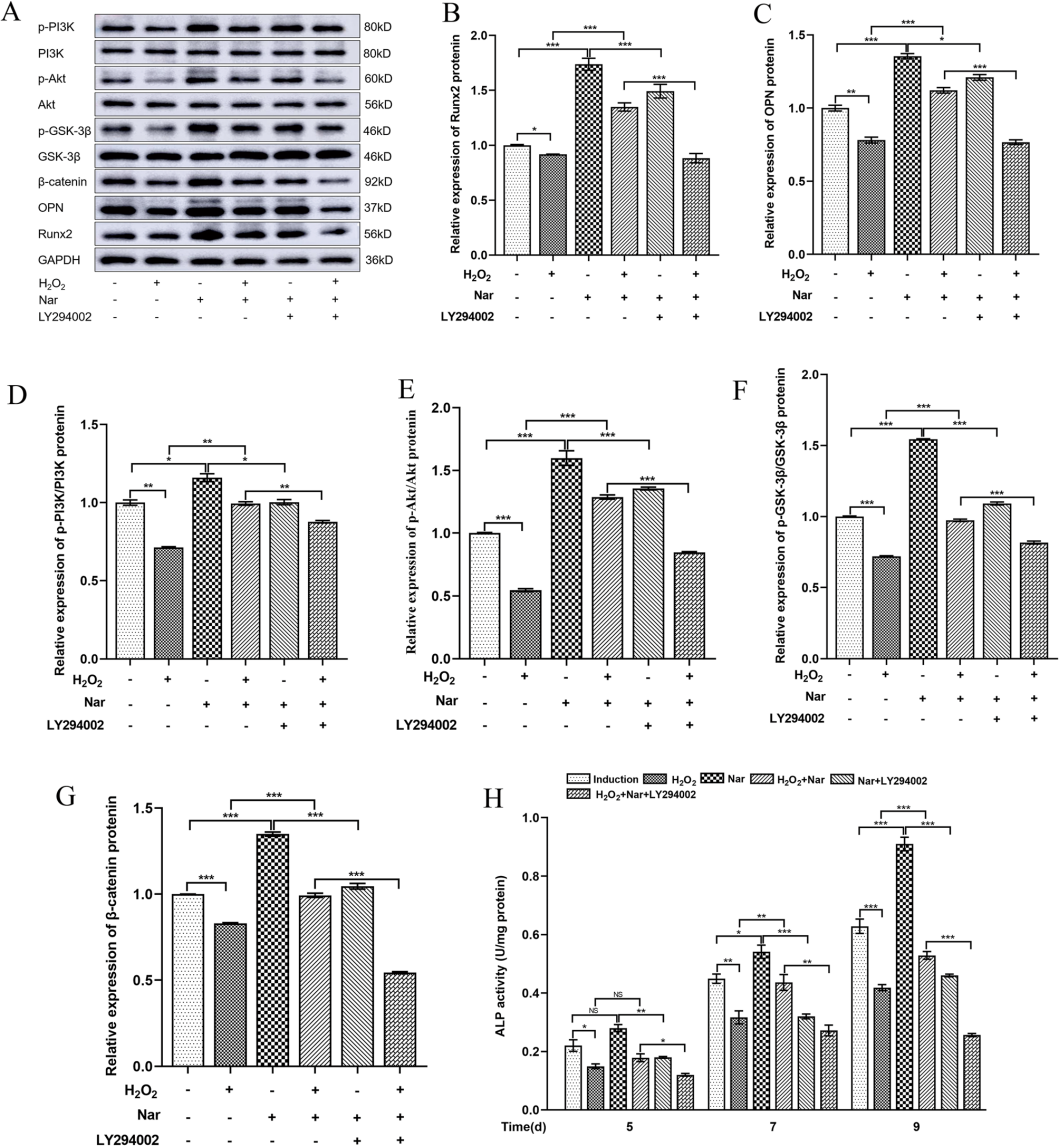
The effect of Nar on the osteogenic differentiation of MC3T3-E1 cells and PI3K/Akt signalling under oxidative stress. (**A**) Protein expression in each group; (**B**–**G**) Relative expression level of Runx2 (**B**), OPN (**C**), p-PI3K/PI3K (**D**), p-Akt/Akt (**E**), p-GSK-3β/GSK-3β (**F**), and β-catenin (**G**). (**H**) ALP activity in each group. *P < 0.05, **P < 0.01, ***P < 0.001 (n = 4).

Changes in the related proteins in each group were detected to investigate the effect of Nar on MC3T3-E1 cell osteoblast differentiation and the PI3K/Akt signalling pathway under oxidative stress. Compared to the induction group, H_2_O_2-_mediated inhibition and Nar-mediated promotion of osteogenic differentiation were related to the PI3K/Akt signaling pathway (Fig. 8). Compared to the H_2_O_2_ group, the expression of related proteins in the H_2_O_2_ + Nar group increased, indicating that Nar promoted the osteogenic differentiation of MC3T3-E1 cells under oxidative stress via the PI3K/Akt signaling pathway (Fig. 8). Compared to the Nar group, the expression of related proteins in the H_2_O_2_ + Nar group decreased after the addition of LY294002, a PI3K/Akt signaling inhibitor, further indicating that Nar promoted the osteogenic differentiation of MC3T3-E1 cells via PI3K/Akt signaling under oxidative stress (Fig. 8). Intracellular ALP activity was measured at 5, 7, and 9 days after osteogenic induction, and the results were consistent with the protein expression levels (Fig. 8H). The differences between all groups were statistically significant (*P* < 0.05).

## Discussion

As the prevalence of OP continues to increase as the population ages, developing effective prevention strategies is urgently required^21^. Oxidative stress is an important mechanism affecting OP pathogenesis that can cause excessive bone loss in the human body by altering osteoblast, osteoclast, and bone marrow stromal cell (BMSC) proliferation and metabolism, resulting in an imbalance in bone homeostasis^22^. Currently, using anti-oxidants to inhibit oxidative stress and delay the development of OP is an important strategy for OP treatment. However, alternative anti-oxidant therapies with improved clinical efficacy and cost-efficiency with fewer side effects are needed to improve patients' quality of life with OP.

This study investigated the specific mechanism of OP pathogenesis by using the GEO2R and R language packages to analyze the GEO dataset comprehensively. A total of 2911 OP-related DEGs then underwent enrichment analyses using KEGG and GO. KEGG analysis revealed that OP-related DEGs were mainly enriched in the PI3K/Akt and Wnt signaling pathways, which was consistent with numerous studies reporting a role for the PI3K/Akt and Wnt signaling in osteogenic differentiation^23,24^. GO function analysis showed that OP-related DEGs were mainly enriched in response to oxidative stress, ROS, and H_2_O_2_ in BP and were mainly enriched in binding growth factor receptors, peroxisome proliferator-activated receptors, and NO synthase in MF. NO is a typical free radical with strong reactivity that can react with superoxide anions to form nitroso complexes, causing high oxidative stress^25^. This coincides with the BP and MF results, which confirmed that oxidative stress caused by excessive ROS production was a major cause of OP and suggested that eliminating ROS may be the key to OP treatment^26^. In addition, the CC results showed that OP-related DEGs were closely related to intercellular junctions and the nuclear envelope.

Among the 29 potential Nar targets found in this study, the following 10 core targets were selected based on degree value: ER1, HSP90AA1, ESR2, ABCG2, DNMT1, CA12, PTPN1, CA9, TUBA1A, and CYP1A2. Upon determining the overlap between OP-related proteins and these core targets, we found 3 OP-related Nar potential targets: ESR1, HSP90AA1, and ESR2. In particular, our findings implicated ESR1 as a key target for Nar's action. Previous studies have discussed the potential targets of Nar and addressed molecular docking between target proteins and drug molecules^27^; however, we further refined these Nar targets and revealed their important roles in the present study. Specifically, molecular docking and protein–ligand interaction analyses revealed that Nar had a good affinity for ESR1 and HSP90AA1 and identified multiple protein–ligand interactions.

ESR activation has been proposed as a positive regulator of the Wnt/β-catenin and PI3K/Akt signaling, with a cross-talk relationship between both pathways reported^28,29^. Since the primary OP is characterized by steroid hormone deficiency and high oxidative stress, and based on the network pharmacology results of this study, we speculated that Nar may regulate Wnt/β-catenin and PI3K/Akt signaling by activating ESR and subsequently reverse oxidative stress-impaired osteogenic differentiation.

This study used bioinformatics to confirm that OP is closely related to oxidative stress and the PI3K/Akt and Wnt signaling pathways. Nar, one of the main components of traditional Chinese medicine, has anti-inflammatory, anti-oxidant, bone-promoting, and other pharmacological properties. The first study to report Nar-mediated promotion of osteogenic differentiation used BMSCs cultured with varying concentrations of Nar^30^. In our previous study, Nar promoted the osteogenic differentiation of human adipose mesenchymal stem cells via the Wnt/β-catenin signaling pathway under oxidative damage conditions, further highlighting its anti-oxidant and osteogenic effects^8^. The results showed that the maturation process of MC3T3-E1 cells could be divided into three stages: cell proliferation, early differentiation (characterized by ALP secretion), and mineralized nodule formation. As these three stages were similar to the differentiation and maturation of normal osteoblasts and were the experimental models for the study of osteoblast maturation^31^, MC3T3-E1 cells were used in the present study to explore further the relationship between Nar-mediated osteogenic differentiation and oxidative stress resistance and its related mechanisms.

Osteoblasts require moderate physical activity for bone formation and remodeling. An increase in osteoblast quantity can be achieved by promoting the proliferation and differentiation of osteoblast progeny cells and reducing the death of mature osteoblasts^32^. The results of our CCK-8 assay showed that 0.1 μmol/L Nar could effectively improve the proliferative activity of MC3T3-E1 cells. However, the viability of MC3T3-E1 cells decreased significantly after treatment with 0.3 mmol/L H_2_O_2_, with a cell survival rate of 75%.

As an early marker of osteoblast differentiation, ALP promotes physiological calcium salt deposition by hydrolyzing glycerophosphate sodium, which plays an important role in mineralization during osteogenesis^33^. In the current study, osteogenesis was induced in MC3T3-E1 cells, and ALP activity, expression of intracellular osteoblast-related proteins, and extracellular calcium salt deposition were measured. The results showed that the osteogenic ability of MC3T3E1 cells decreased under oxidative stress, while Nar could improve the osteogenic differentiation inhibited by oxidative stress to a certain extent.

Wnt/β-catenin signaling pathway is a classical pathway that determines cell proliferation and differentiation and influences cell function by regulating β-catenin levels and subcellular localization^34^. Without the 6Wnt ligand, β-catenin is phosphorylated at its NH_2_ terminal and degraded by a multiprotein complex comprising GSK-3β, APC, and Axin's skeleton protein^35,36^. Activation of the Wnt pathway prevents the formation of this multiprotein complex and subsequent β-catenin degradation^36^. Instead, it accumulates and translocates to the nucleus to activate the transcription of a series of genes. This study found that under oxidative stress, Nar increased the expression of the osteogenic proteins Runx2 and OPN, and Wnt/β-catenin signalling-related proteins, while Wnt-C59 inhibited this effect. These results suggest that Nar promotes the osteogenic differentiation of MC3T3-E1 cells inhibited by oxidative stress, potentially via the Wnt/β-catenin signaling pathway.

The PI3K signal transduction process is an important pathway in regulating cell proliferation, differentiation, and apoptosis^37^. In a previous study, H_2_O_2_ was used to establish a model of oxidative stress in BMSCs and explored the effects of ebselen treatment. The results showed that ebselen-promoted osteogenic differentiation was inhibited by oxidative stress via the PI3K/Akt signaling pathway^38^. However, it has not yet been reported whether Nar, with its strong anti-oxidant properties, can similarly promote oxidative stress-impaired osteogenic differentiation. In the current study, Nar increased the expression of PI3K/Akt signaling proteins related to osteogenic differentiation in MC3T3-E1 cells with oxidative stress injury, and this effect was blocked by the PI3K/Akt signaling inhibitor, LY294002. Therefore, Nar may promote osteogenic differentiation inhibited by oxidative stress via the PI3K/Akt signaling pathway.

This study had several limitations. First, our hypothesis was only tested in vitro and needs to be further verified in vivo. Second, whether and how Wnt/β-catenin and PI3K/Akt signaling interact remains unclear and warrants further exploration.

In conclusion, this study explored how Nar promotes osteogenic differentiation by establishing an oxidative stress model using H_2_O_2_ and evaluating Wnt/β-catenin and PI3K/Akt signalling-related proteins in OP samples. Notably, Nar could induce the phosphorylation of GSK-3β by activating Wnt/β-catenin and PI3K/Akt signaling, leading to the accumulation of β-catenin, and ultimately activating the expression of osteogenic markers. These results suggest that Nar promotes the osteogenic differentiation of MC3T3-E1 cells inhibited by oxidative stress by activating Wnt/β-catenin and PI3K/Akt signaling pathways and presents Nar as a potential treatment for OP. Owing to the development of traditional Chinese medicine and its increasing use in modern medicine, we expect breakthroughs that will establish an effective clinical treatment strategy for OP management and improve patients' quality of life soon.

## Materials and methods

### Data collection and processing

Using the Gene Expression Omnibus (GEO) database (https://www.ncbi.nlm.nih.gov/GEO/) with the keyword ‘osteoporosis’ for retrieval, we downloaded the GSE35958^39^ dataset for OP and non-OP samples. Using a standardized GEO2R gene microarray (http://www.ncbi.nlm.nih.Gov/geo/geo2r), we compared the DEGs between both samples and defined those that had a |logFC (fold change)|> 1 and false discovery rate − log10(*P* value) < 1.3 (FDR < 0.05) as OP-related DEGs. The data were visualized using R 4.3.2’s ggplot2 3.5.0 and pheatmap 1.0.12 packages (R Core Team, Austria).

### Enrichment analysis of DEGs

The General Database for Annotation, Visualisation, and Integrated Discovery^40^ (DAVID; https://david.ncifcrf.gov) was used for Gene Ontology (GO) and Kyoto Encyclopaedia of Genes and Genomes^41^ (KEGG) functional enrichment analysis. The GO analysis consisted of three components: biological process (BP), molecular function (MF), and cellular component (CC). The data were visualised using the ggplot2 3.5.0 package. *P*-values < 0.05 were considered statistically significant.

### Screening of Nar core targets

“Naringin” was used as a keyword in PubChem (https://pubchem.ncbi.nlm.nih.gov/) to retrieve the Simplified Molecular Input Line Entry System (SMILES) chemical formula. Then, using this formula, we screened the TargetNet database (http://targetnet.scbdd.com/home/index/) and only included models with an area under the curve (AUC) cut-off value of 0.7. The potential targets of Nar were identified using extended-connectivity (ECFP4) fingerprint retrieval, and a protein–protein interaction (PPI) network was drawn using the Search Tool for the Retrieval of Interacting Genes/Proteins (STRING) database. The degree value of each node in the PPI network was sorted using Cytoscape_v3.10.0-SNAPSHOT^42^ (Cytoscape Team, USA). The top 10 targets with the highest scores were selected and defined as the core targets.

### Screening of the potential OP-associated drug targets

OP-related proteins were searched in Comparative Toxicogenomics Database (CTD), DisGeNET, and GeneCards using “osteoporosis” as the keyword. The core target intersection was determined using an online Venn diagram tool (http://bioinformatics.psb.ugent.be/webtools/Venn/), and the potential Nar action targets associated with OP were obtained.

### Molecular docking

Oestrogen receptor 1 (ESR1) and heat shock protein 90 alpha family class A member 1 (HSP90AA1) proteins obtained from the Protein Data Bank (PDB) (https://www.rcsb.org/) were pre-treated for structural optimization using molecular operating environment software (MOE; Chemical Computing Group, Canada) and the sitefinder function was used to identify the binding pockets For molecular docking, the receptor was set as ESR1 or HSP90AA1, site as site 1, and the ligand as Nar. The results of the analysis were visualized using PyMOL 2.6.0 software^43^ (Schrödinger, Inc., USA).

### Cell culture

Resuscitated MC3T3-E1 cells (EK-Bioscience, China) were cultured in minimum essential medium alpha (α-MEM; Shanghai BasalMedia Technologies Co., Ltd., China) medium containing 10% fetal bovine serum (Nanjing SenBeiJia Biological Technology Co., Ltd., China) and 1% penicillin–streptomycin solution (Nanjing SenBeiJia Biological Technology Co., Ltd., China) at 37℃ in a 5% CO_2_ incubator (Sanyo, Japan). After the cells reached 80%–90% confluency, they were treated with 0.25% trypsin/0.02% ethylenediaminetetraacetic acid solution (Sigma, USA) and re-plated at a dilution of 1:3.

### CCK-8 assay

MC3T3-E1 cells in the logarithmic growth phase were seeded in 96-well plates with 1 × 10^4^ cells/well and cultured for 24 h. Cells were treated with a low-serum medium containing various Nar concentrations (Sigma, USA) or H_2_O_2_ (Sigma, USA) for 24 h or with Nar following 24 h of H_2_O_2_ treatment for 24, 48, and 72 h. Cell counting kit-8 (CCK-8) reagent (Meilun Biological, China) was added to the cells and incubated for 2 h. Absorbances were measured at 450 nm using a microplate reader (Molecular Devices, USA). Cell survival rate was calculated using the optical density (OD) values as follows:

$$\begin{gathered} \left[ {\left( {OD\; value\; of\; experimental\; wells} \right.} \right. \hfill \\ - \left. {OD\; value\; of\; blank\; wells} \right)/\left( {OD\; value\; of\; control\; wells} \right. \hfill \\ - \left. {\left. {OD\; value \;of \;blank\; wells} \right)} \right] \times 100\% . \hfill \\ \end{gathered}$$

### Cytotoxicity assay

MC3T3-E1 cells were subjected to 0.3 mM H_2_O_2_ and 0.1 µM Nar for 24 h to detect lactate dehydrogenase (LDH) (Elabscience, China) production. The MC3T3-E1 cells were seeded in 96-well plates and cultured for 24 h. Cells were treated according to the following groups: control, induction, H_2_O_2_, Nar, and H_2_O_2_ + Nar. Then, 10 μL of lysate was added to the control well with maximum enzyme activity, and the cells were cultured for another 1 h after blowing and mixing. After centrifugation at 400 × *g* for 5 min, 50 μL of supernatant to be measured and 50 μL of reaction working solution was added to each well, and the microplate was shaken. After incubating at 37 °C for 10 min, the reaction termination solution was added, and the absorbance was measured at 450 nm using a microplate reader. Cytotoxicity was calculated as follows:

$$\begin{gathered} \left[ {\left( {D\; value\; of\; sample\; test\; well} \right.} \right. \hfill \\ - \left. {OD\; value\; of\; sample\; control\; well} \right) \hfill \\ /\left( {OD\; value\; of\; maximum\; enzyme\; activity\; control\; well\;} \right. \hfill \\ - \left. {\left. {OD\; value\; of\; sample\; control\; well} \right)} \right] \times 100\% \hfill \\ \end{gathered}$$

### ROS assay

After cleaning with phosphate-buffered saline (PBS), a 2'-7'dichlorofluorescin diacetate (DCFH-DA) (Applygen Technologies, China) probe diluted with α-MEM (final concentration of 10 μM) was added to the MC3T3-E1 cells in 6-well plates and incubated at 37 °C for 30 min in the dark. During this period, the probe was gently shaken every 5 min to ensure full contact with the cells. The cells were washed three times and then re-suspended with α-MEM. ROS levels were recorded under a fluorescence microscope within 30 min at 525 nm to determine the establishment of the oxidative stress model. Fluorescence staining used ImageJ 13.0.6 software^44^ to detect fluorescence brightness as ROS expression. The cell suspension was detected by fluorescent enzyme, the absorbance was measured at 520 nm, and the results were expressed by fluorescence value.

### Nitric oxide assay

Dilute the standard in the kit to 800, 400, 200, 100, 50, 25, 12.5, 6.25, 3.13, 1.57 μM, the OD values of each group were detected by microplate reader, and the concentration calculation formula was obtained by making a standard curve. In 96-well plate, 50 μL control(10%FBS-α-MEM) and 50 μL Griess reagent were added and incubated at room temperature in the dark for 5 min. Then, the absorbance was detected using at 540 nm. A standard curve was drawn, the OD values of the sample group were substituted into the formula to calculate the NO expression in the group, and the concentration of NO was calculated to confirm the establishment of the oxidative stress model.

### Alkaline phosphatase staining

Osteogenesis was induced in the MC3T3-E1 cells seeded in 6-well plates according to the different groups. Cell culture was performed with α-MEM in the control group, with basic osteogenic differentiation medium (α-MEM, 10% FBS, 50 μg/mL ascorbic acid, and 10 mM sodium β-glycerophosphate) in the induction group, and in the hydrogen peroxide group, basic osteogenic differentiation medium was added after 24 h of H_2_O_2_ action. For the Nar group, we added Nar to the basal osteogenic differentiation medium, while for the H_2_O_2_ + Nar group, we added the above Nar-containing osteogenic differentiation medium after 24 h of H_2_O_2_ action. and stained using the BCIP/NBT alkaline phosphatase chromogenic kit (Beyotime, China). After 2 weeks, the medium was removed, and the cells were washed three times with PBS for 3–5 min each, fixed with 4% paraformaldehyde for 15 min, and washed a further three times with PBS. Then, 1 mL of the staining working solution was added to each well and incubated at room temperature for 20 min in the dark. After removing the working solution, the staining solution was washed three times with double distilled H_2_O and imaged under an inverted microscope.

### Alizarin red S staining

Osteogenesis was induced in the MC3T3-E1 cells seeded in 6-well plates according to different groups and then stained with the osteoblast mineralized nodule staining kit (Beyotime, China). After 21 days, the medium was discarded, and the cell surface was lightly washed three times with PBS for 3–5 min each, fixed with 4% paraformaldehyde for 15 min, and washed a further three times with double distilled H_2_O. Alizarin Red S staining working solution (1 mL) was added to each well and the cells were stained for 30 min. After discarding the solution, the cells were washed three times with ddH_2_O and imaged under an inverted microscope.

### Alkaline phosphatase activity assay

Osteogenesis was induced in MC3T3-E1 cells in 6-well plates for 5, 7, and 9 days, according to different groups. The cells were harvested and lysed, and the remaining supernatant was collected. The alkaline phosphatase (ALP) detection kit (Beyotime, China) was used following the manufacturer’s instructions. Absorbances were measured at 405 nm, and the activity of ALP was calculated (Table S1).

### Western blot

Osteogenesis was induced in the MC3T3-E1 cells for 14 days, and they were treated according to different groups. Total protein was extracted using radioimmunoprecipitation assay lysis buffer (Beyotime, China), quantified using the bicinchoninic acid method (Applygen Technologies Inc., China), and denatured by boiling after adding loading buffer. Whole-cell protein extracts were separated by sodium dodecyl sulphate–polyacrylamide gel electrophoresis and analyzed using Western blot (WB). The amount of protein was loaded with 10 μg/well. The PVDF membranes (0.22 μm) were then blocked with 5% BSA for 2 h. The following primary antibodies were prepared at 1:1000 after washing the membrane and incubated overnight at 4 °C: anti-GAPDH (Affinity, USA), anti-PI3K (Affinity, China), anti-p-PI3K (Affinity, China), anti-Akt (Affinity, China), anti-p-Akt (Affinity, China), anti-GSK-3β (Affinity, China), anti-p-GSK-3β (Affinity, China), anti-LRP-5 (Abcam, China), anti-β-catenin (Cell Signalling Technology, USA), anti-Runx2 (Affinity, China), and anti-OPN (Beyotime, China). The membranes were then washed three times with PBS-Tween, incubated with a secondary antibody for 2 h at room temperature, and washed three more times. Enhanced chemiluminescence solution (Applygen Technologies, China) was then applied, and the relative image intensity was calculated using ImageJ software (Table S1).

### Statistical analysis

Data are expressed as the mean ± standard deviation. All statistical analyses were performed using SPSS 25.0 (IBM Corp., USA) and GraphPad Prism 8.0 (GraphPad Software, Inc., USA). The independent sample t-test was used for comparison between two groups. A one-way analysis of variance (ANOVA) followed by Tukey’s post-hoc test was used to compare multiple groups. *P*-values < 0.05 were considered statistically significant.

### Ethics approval and consent to participate

The data for the bioinformatics analyses in this study were obtained from online databases, and the cell lines were purchased by the company, so no ethical approvals were involved.

### Supplementary Information

Supplementary Table S1.

## Data Availability

The data during the current study are available from the corresponding author, H.W., upon reasonable request.
